# 1-L Transcription of SARS-CoV-2 Spike Protein S1 Subunit

**DOI:** 10.3390/ijms25084440

**Published:** 2024-04-18

**Authors:** Jozef Nahalka

**Affiliations:** 1 Institute of Chemistry, Centre for Glycomics, Slovak Academy of Sciences, Dubravska Cesta 9, SK-84538 Bratislava, Slovakia; nahalka@savba.sk; 2 Institute of Chemistry, Centre of Excellence for White-Green Biotechnology, Slovak Academy of Sciences, Trieda Andreja Hlinku 2, SK-94976 Nitra, Slovakia

**Keywords:** COVID-19, SARS-CoV-2 spike protein S1 subunit, protein–RNA recognition, post-transcriptional regulations, vaccination against SARS-CoV-2 infection

## Abstract

The COVID-19 pandemic prompted rapid research on SARS-CoV-2 pathogenicity. Consequently, new data can be used to advance the molecular understanding of SARS-CoV-2 infection. The present bioinformatics study discusses the “spikeopathy” at the molecular level and focuses on the possible post-transcriptional regulation of the SARS-CoV-2 spike protein S1 subunit in the host cell/tissue. A theoretical protein–RNA recognition code was used to check the compatibility of the SARS-CoV-2 spike protein S1 subunit with mRNAs in the human transcriptome (1-L transcription). The principle for this method is elucidated on the defined RNA binding protein GEMIN5 (gem nuclear organelle-associated protein 5) and RNU2-1 (U2 spliceosomal RNA). Using the method described here, it was shown that 45% of the genes/proteins identified by 1-L transcription of the SARS-CoV-2 spike protein S1 subunit are directly linked to COVID-19, 39% are indirectly linked to COVID-19, and 16% cannot currently be associated with COVID-19. The identified genes/proteins are associated with stroke, diabetes, and cardiac injury.

## 1. Introduction

**Figure 1:**
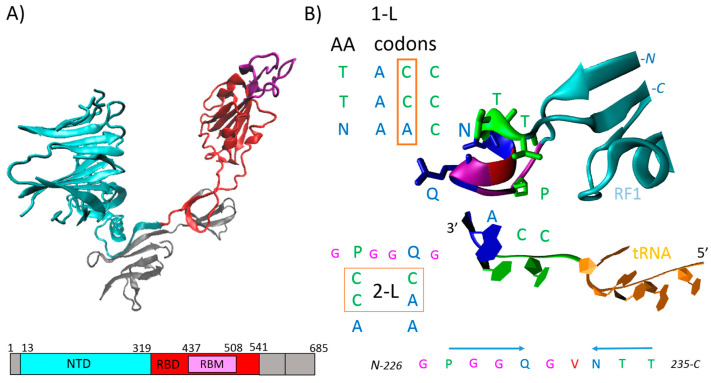
(**A**) SARS-CoV-2 spike protein S1 subunit in the prefusion conformation and its schematic domain representation. NTD, N-terminal domain (cyan); RBD, receptor-binding domain (red); RBM, receptor-binding motif (magenta). (**B**) The ribosomal release factor 1 (RF1) interacting with P-site tRNA (4V63). 1-L, one-letter code—second nucleotide in codons; 2-L, two-letter code—first two nucleotides in codons [16]. At a shorter distance, proline 227 (P) and glutamine 230 (Q) recognize terminal CCA3′ nucleotide sequence using the 2-L code; at a longer distance, asparagine 233 (N), threonine 234 (T) and T235 recognize terminal CCA3′ nucleotide sequence using 1-L code in the reversed mode. Interestingly, these two sequences are spaced with glycine (G) and valine (V), which can be 2-L transcribed to GGU3′ complementary sequence. The coordinates were downloaded from Protein Data Bank, 6VYB (**A**) and 4V63 (**B**) are the corresponding PDB codes, and Visual Molecular Dynamics (VMD 1.9.3) was used for the visualization.

Vaccines against the coronavirus SARS-CoV-2 have been given to billions of people to protect them from COVID-19. The vaccines developed by Pfizer–BioNTech and by Moderna consist of an mRNA-modified version of spike protein packaged in a fatty nanoparticle [1]. The AstraZeneca ChAdOx1 vaccine is based on a replication-incompetent adenoviral vector that delivers the gene for the spike protein [2]. SARS-CoV-2 vaccines drive expression of the spike protein, as this structural protein is the main target of antibody-producing B cells. During viral infection, the spike protein is cleaved into S1 and S2 subunits, with S1 serving the function of receptor-binding and S2 the function of membrane fusion. The immunogenicity of the S1 subunit is much higher than that of the S2 subunit. S1 is better exposed and is targeted by some of the body’s most potent infection-blocking antibodies [1]. Future coronavirus vaccine development is likely to focus more on S1, with the receptor-binding domain (RBD) especially being a region of interest (Figure 1A). The RBD binds to the ACE2 receptor in human cells [1], and an RBD tandem dimer fused to the S1 N-terminal domain (NTD) was recently proposed [3]. Nanoparticles dotted with RBDs from SARS-CoV-2 and coronaviruses from the same family, i.e., ‘mosaic’ vaccines, could be used as next-generation vaccines. However, market leaders adapt their existing vaccines very quickly, as shown by the rapid development of bivalent vaccines that included an Omicron component [1]. In summary, vaccines against the SARS-CoV-2 coronavirus are based on the spike protein or its components, and this is likely to continue into the foreseeable future. 

Cases of apparent secondary immune thrombocytopenia after vaccination with both the Pfizer and Moderna vaccines have been reported and reached public attention during the first vaccination campaign [4]. Furthermore, the occurrence of thrombocytopenic, thromboembolic, and hemorrhagic events after vaccination with the AstraZeneca ChAdOx1 vaccine led some countries to restrict its use [5]. Data from the vaccine adverse event reporting system (VAERS) associated the Pfizer-BioNTech and Moderna vaccines with myocarditis and anaphylaxis, and the Johnson & Johnson vaccine with thrombosis with thrombocytopenia syndrome and Guillain-Barré syndrome [6]. Cardiovascular [7] and neurological [8] complications following COVID-19 vaccination have been linked to toxicity from the SARS-CoV-2 spike protein, with the term ‘spikeopathy’ recently introduced [9]. In summary, more research is needed on spike toxicity from SARS-CoV-2 but also from the spike protein produced by mRNA and DNA vaccines.

This bioinformatics study focuses on possible post-transcriptional regulation of the SARS-CoV-2 spike protein S1 subunit in host cells/tissues. Ten regulated genes were previously identified by 1-L transcription [10] in relation to protein–RNA post-transcriptional regulation of the S1 subunit: PARG (poly[ADP-ribose] glycohydrolase), DAW1 (dynein assembly factor with WD repeats 1), ZYG11B (zyg-11 family member B, cell cycle regulator), PIKFYVE (phosphoinositide kinase, FYVE-type zinc finger containing), RABEP1 (rabaptin, RAB GTPase binding effector protein 1), MSRA (methionine sulfoxide reductase A), PLB1 (phospholipase B1), TJP2 (tight-junction protein 2), ZEB2 (zinc finger E-box binding homeobox 2), and XYLB (xylulokinase B). As confirmed experimentally by others, these genes/proteins are important during SARS-CoV-2 infection. For example, the PIKFYVE inhibitor XMU-MP-7 effectively overrides SARS-CoV-2 and its variants, including delta and Omicron [11]. In another example, virally enhanced CUL2-ZYG11B activity leads to increased ubiquitination and subsequent proteasome-mediated degradation of IFT46 (intraflagellar transport 46), thereby impairing both the biogenesis and maintenance of cilia [12]. In summary, the profile of genes identified to date gives credence to the methodological pipeline used to identify these genes (1-L transcription). Therefore, the same approach was used here to identify another 36 genes/proteins that are potentially involved in ‘spikeopathy’.

1-L transcription is a very simple method that was introduced recently [10,13,14,15] and which applies a theoretical protein–RNA recognition code (Figure 1B) [16]. As shown in the figure for ribosomal release factor 1 (RF1) and tRNA, the primary protein structures are involved in protein–RNA recognition/interaction processes. In this case, the CCA3′ end of the tRNA is recognized by proline (CCA codon) and glutamine (CAA codon) inserted into polyglycine (2-L code), and two threonine (ACC codon) residues and one asparagine (AAC codon) residue (1-L code) in the reverse mode (Figure 1B). The 1-L transcription method is based on the principle that RNA binding proteins (RBPs) use at least one amino acid sequence that can be 1-L transcribed into the exact nucleotide sequence of the recognized RNA. An example that demonstrates the procedure will be given later for the defined RBP GEMIN5 (gem nuclear organelle-associated protein 5) and RNU2-1 (U2 spliceosomal RNA).

In summary, vaccines against coronavirus SARS-CoV-2 are likely to continue to be based on the spike protein. However, toxicity from the SARS-CoV-2 spike protein is associated with cardiovascular and neurological complications. “1-L transcription” of the SARS-CoV-2 spike protein S1 subunit and subsequent BLASTn comparison of the transcripts with the human transcriptome have revealed novel genes/proteins that are potentially involved in ‘spikeopathy’. These novel genes were comprehensively reviewed, and their functions and profile were found to be consistent with ‘spikeopathy’ and post-COVID-19 syndrome [9,17]. 

## 2. Results

The randomly selected RBP GEMIN5 demonstrates the methodology in Section 2.1. Using the methodology for the SARS-CoV-2 spike protein S1 subunit and reviewing the literature, it was found that PARG, BCAP29, ZYG11B, USP46, ZNF385D, PIKFYVE, ADGRL4, DSC3, NECTIN2, CPSF2 (Section 2.2) and XYLB, FILIP1L, GAS2, BCL2L13, WNK3, STK39, COPB2, N4BP2L2, PTPN20, and WWP1 (Section 2.3) identified genes/proteins are directly linked to SARS-CoV-2 infection (discussed in Section 3.2.1); RABEP1, LSAMP, NUDCD2, XXYLT1, PRKAA2, SETBP1, PPP1R26 (Section 2.2) and MYCT1, PLB1, GRB14, BMPR2, TJP2, ZEB2, MSRA, UNC79, LRPPRC, USP24 (Section 2.3) are indirectly linked to SARS-CoV-2 infection (discussed in Section 3.2.2); and DAW1, ESF1, KCTD2 (Section 2.2), USP45, PNMA8A, SYNDIG1L, and CC2D2B (Section 2.3) are novel genes/proteins that may be linked to COVID-19. The identified genes/proteins are associated with immune responses and inflammation (discussed in Section 3.3), diabetes and cardiac stress (discussed in Section 3.4), and cilia and lung injury (discussed in Section 3.5). 

### 2.1. GEMIN5 as an Example of How Protein Primary Structure Is Involved in Protein–RNA Recognition

An example of the 1-L transcription procedure presented here is for the defined RBP GEMIN5 (gem nuclear organelle-associated protein 5) and RNU2-1 (U2 spliceosomal RNA). GEMIN5 participates in the SMN complex, which drives structural changes in human spliceosomal small-nuclear RNAs (snRNAs) to enable the assembly of small-nuclear ribonucleoproteins (snRNPs) [18]. At least five different kinds of snRNPs join the spliceosome to participate in splicing. The SMN complex consists of nine proteins, namely SMN (survival motor neuron), GEMIN2 to GEMIN-8, and UNRIP [19]. GEMIN3 is the essential helicase within the complex, while GEMIN5 acts as the essential “identifier” of the RNA substrate and recognizes the Sm site and binding pre-snRNAs [18]. The N-terminal WD40 domain of GEMIN5 is responsible for binding the Sm site inside the pre-snRNA [20]. The C-terminal region plays an important role in regulating translation by binding directly to mRNAs. It is also responsible for the high-order structural architecture, consisting of a dimer of pentamers [21]. In summary, GEMIN5 is a regular RBP that recognizes the Sm site and binds pre-snRNAs.

**Figure 2:**
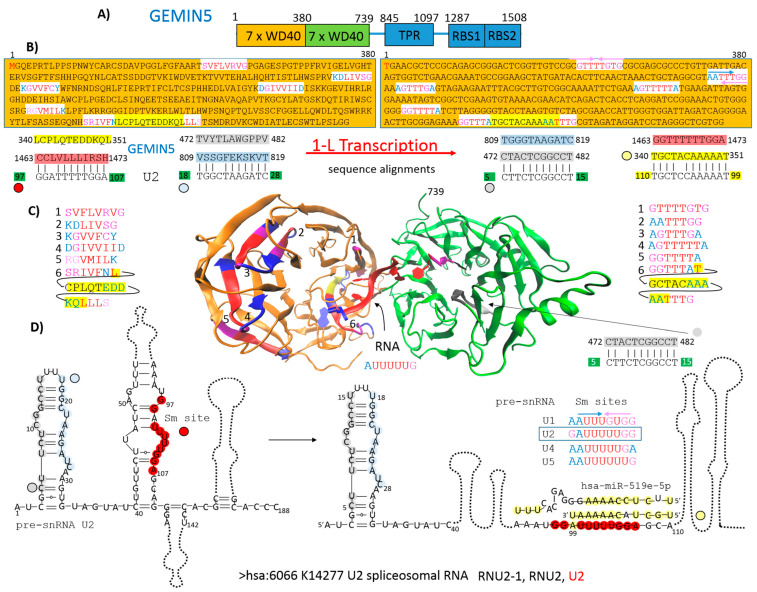
GEMIN5 as a sample example of how protein primary structure is involved in protein–RNA recognition. (**A**) Schematic domain architecture of full-length human GEMIN5 protein. The N-terminal domain of WD40 contains 14 WD40 repeats that form two seven-bladed β propellers (WD1 in orange and WD2 in green). TPR domain, the tetratricopeptide repeat. RBS1 and RBS2, non-canonical RNA binding sites. (**B**) 1-L Transcription. Amino acid sequence on the left, nucleotide sequence obtained by 1-L transcription on the right. Identified regions of similarity in alignment with RNU2-1 RNA are shown on both sides. Regions are marked/highlighted in different colors. (**C**) Crystal structure of N-terminal domain (amino acids 1–739) in complex with the Sm site RNA (5GXH). Amino acid sequences colored in the structure are on the left and 1-L compatible nucleotide sequences are on the right. The amino acid sequence of 1-L compatible with the reverse complement RNA sequence of the U2 Sm site (plus/minus strand) is highlighted in yellow. (**D**) Indication of pre-snRNA U2 folding pathway (graphically abstracted from [18]) and highlighting of 1-L compatibility with GEMIN5 primary structure.

To demonstrate 1-L transcription and how the protein primary structure is involved in protein–RNA interactions, the entire amino acid sequence of GEMIN5 was transcribed into a nucleotide sequence. Serine was transcribed as guanosine, and only the *N*-(AA)n-*C* direction was applied. The resulting nucleotide sequence was aligned with the nucleotide sequence of RNU2-1, with an expected threshold of 5 used to minimize the number of hits. The analysis revealed four sequences in GEMIN5 that are compatible with RNU2-1 (Figure 2B). Two sequences are exactly compatible with stem 1 and two with the Sm site (Figure 2D). In the U2 pre-snRNA folding pathway, stem 1 does not change, but the Sm site is exposed [18]. GEMIN5 has 1-L compatibility to both the U2 Sm site sequence (strand plus/plus) and to the U2 Sm site reverse complement RNA sequence (strand plus/minus) (Figure 2B). The N-terminal WD40 domain is reportedly sufficient to bind the pre-snRNA Sm site [20]. Indeed, a primary structure compatible with the reverse complementary RNA sequence of the U2 Sm site (plus/minus strand) is located inside the WD40 domain (340 LCPLQTEDDKQL/TGCTCCAAAAAAAT 351). However, the primary structure of GEMIN5 compatible with the U2 Sm site (strand plus/plus) is located at the C-terminus (1463 CCLVLLLIRSH/GGTTTTTTGGA 1473). The longer recognition primary structures in RBPs are usually conserved at the N or C terminus, or in disordered regions, but reading of the Sm site by the WD domain is actually performed by different 3–5-amino-acid sequences and in both directions (Figure 2B,C). For example, 335RIVFN/GUUUA339 is capable of reading the AUUUUUG Sm site. It reads AUUU in the *C*-(AA)n-*N* direction and GUUU in the *N*-(AA)n-*C* direction (Figure 2B,C). The use of a BLASTn word size of 7 reveals only one recognition sequence in the AUUUUUG Sm site at the C-terminus of GEMIN5. The 1-L transcription method is based on the assumption that RNA binding proteins (RBPs) use at least one amino acid sequence that can be 1-L transcribed into the exact nucleotide sequence of the recognized RNA. This can be used to identify the relatedness between proteins and RNA. 

### 2.2. Genes/Proteins Identified by 1-L Transcription of N-(AA)n-C Sequences 

**Figure 3:**
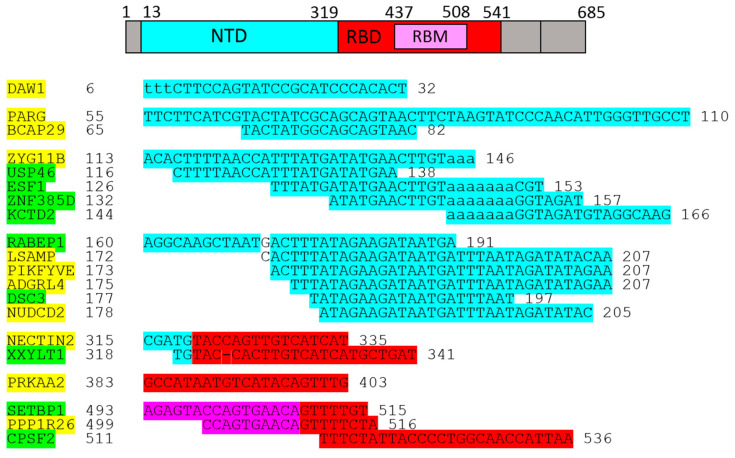
Identified genes/proteins through 1-L transcription of *N*-(AA)n-*C* sequence. Schematic domain architecture of S1 subunit sequence. Green highlights show alignments with the gene transcript RNA sequence (post-transcriptionally repressed); yellow highlights show alignments with the reverse complement RNA sequences (post-transcriptionally promoted).

As shown above, 1-L transcription and BLASTn alignments lead to both the mRNA sequence (plus/plus strand) and the reverse complementary RNA sequence (plus/minus strand) (Figure 2B). A protein sequence that is 1-L compatible with a reverse complementary RNA sequence is considered herein as a sequence designed to recognize regulatory miRNAs. Micro-RNAs (miRNAs) are small endogenous RNAs that pair and bind to mRNA sites to induce post-transcriptional repression (Figure 2D, has-miR-519e-5p). Reducing the level of miRNAs or other small regulatory RNAs can, therefore, promote translation. Alignments with reverse complement RNA sequences (plus/minus strand) are considered to be promotive (yellow in the figures). In contrast, alignments with the RNA sequence of the gene (strand plus/plus) are considered to be repressive (green in the figures), since the sequestering and blocking of free mRNA by the test protein represses translation. The genes identified according to these alignments are shown in Figure 3.

DAW1 (dynein assembly factor with WD repeats 1, ODA16, WDR69) is a ~50 kDa WD40-repeat protein that is conserved in many organisms with motile cilia. It is a cargo adapter that promotes the import of dynein arms [22]. The transport of ciliary proteins and complexes requires adapters that link them to intra-flagellar transport (IFT) trains. Outer dynein arm 16 (ODA16) physically bridges ODA proteins with the IFT-B complex protein IFT46 [22]. DAW1 appears to regulate the timing for the onset of cilia motility [23].

PARG (poly[ADP-ribose] glycohydrolase, PARG99) possesses both endo- and exo- glycohydrolase activity. It preferentially performs the latter by binding to the two most distal ADP-ribose residues within the poly(ADP-ribose) chain (PAR). By hydrolyzing the ribose–ribose bonds present in PARylted proteins, PARG reverses the action of poly(ADP-ribose) polymerases (PARPs) [24]. Nsp3 is a multi-domain, non-structural SARS-CoV-2 protein with a conserved Mac1 domain. It has mono-ADP-ribose (MAR) and PAR hydrolase activity [25] required for IFN antagonism and efficient virus replication [26,27]. ADP ribose is a product of PARG and Nsp3 activities. It binds to and may over-activate TRPM2 channels, which can lead to intracellular Ca^2+^ overload and a form of programmed cell death [28].

BCAP29 (B cell receptor-associated protein 29) interacts with BAP31 to regulate its function towards some clients (e.g., MHC class I). BAP31 acts as a broad-specificity membrane protein chaperone, with multiple roles in metabolism and the immune system. Several viruses, including SARS-CoV-2, have been found to target BAP31, thereby promoting their survival or life cycle progression [29]. BAP29 is 50% identical to BAP31 and has the same predicted domain organization but without the caspase cleavage sites [29]. A mendelian randomization analysis of the association between SARS-CoV-2 infection and blood constituents found consistent evidence that COVID-19 is causally associated with BCAP29 [30].

ZYG11B (zyg-11 family member B, ZYG11) is a substrate receptor for Cullin 2-RING E3 ubiquitin ligase (CUL2). CUL2 plays an important role in protein quality control and recognizes small N-terminal residues for degradation [31]. SARS-CoV-2 ORF10 was shown to increase the overall E3 ligase activity of the CUL2^ZYG11B^ complex by interacting with ZYG11B. Enhanced CUL2^ZYG11B^ activity by ORF10 causes increased ubiquitination and subsequent proteasome-mediated degradation of IFT46, thereby impairing both the biogenesis and maintenance of cilia [12,32]. 

USP46 (ubiquitin-specific peptidase 46) is a de-ubiquitylase that forms a complex with UAF1 and WDR20 (USP46 complex) to function as a positive regulator of canonical Wnt signaling. The USP46 complex blocks ubiquitylation/degradation of the Wnt co-receptor LRP6 [33]. The Wnt signaling pathway in host cells is often implicated in bacterial and viral infections. This pathway is active in pulmonary epithelial cells, although SARS-CoV-2 infection appears to be insensitive to Wnt inhibitors [34]. USP46 was one of the genes identified by machine learning methods during sequential vaccination with ChAdOx1/BNT162b2 [35].

ESF1 (ESF1 nucleolar pre-rRNA-processing protein homolog) is a ribosome biogenesis protein that links ribosome biogenesis with neural crest cell development and is essential for pharyngeal cartilage formation [36]. ESF1 was also identified as one of five hub genes responsible for obesity-induced cardiac injury by affecting angiogenesis in the heart [37].

ZNF385D (zinc finger protein 385D, ZNF659) is a transcription factor (TF) expressed in systemic–venous endothelial cells [38] and which may be an upstream regulator of MYBL2 [39]. Both ZNF385D and MYBL2 are amongst the hub genes identified to explain the impact of COVID-19 on ischemic stroke [40,41]. MYBL2 was listed in the context of neutrophil degranulation during COVID-19 [42], while ZNF385D was identified in an epigenome-wide association study (EPICOVID) [43].

KCTD2 (potassium channel tetramerization domain containing 2) is a strong interactor with the Gβγ-dimer. G protein–coupled receptors (GPCRs) initiate an array of intracellular signaling programs by activating heterotrimeric G proteins (Gα, Gβ, and Gγ subunits). The Gβγ dimer mediates sensitization of adenylyl cyclase 5 (AC5), which is blunted by KCTD2 binding to Gβγ [44]. The inhibition of AC5 has potential therapeutic applications, not only for cardiac stress but also for aging, diabetes, and obesity [45]. Unfortunately, the repression of KCTD2 has the opposite effect. KCTD2 works as an adaptor for Cullin3 E3 ubiquitin ligase to mediate the polyubiquitination/degradation of c-Myc [46]. c-Myc is a TF with a central role in cell proliferation, glycolytic metabolism, differentiation, and apoptosis.

RABEP1 (rabaptin, RAB GTPase binding effector protein 1) is a regulator of early endosome function. As such, it is an essential mediator of selective autophagy following endosomal injury by chemical- or bacterial-induced membrane damage [47]. RABEP1 is an extended coiled-coil protein with binding sites for RAB4 and RAB5, and for the clathrin coat adaptors AP-1 and GGA. It also forms a stable complex with RABGEF1, which is the GDP/GTP exchange factor for RAB5 [47]. RAB5 regulates the formation of clathrin-coated vesicles (CCVs), the fusion of CCV with early endosomes, and homotypic fusion between early endosomes. RAB7 regulates the transformation of early endosomes to late endosomes by a process referred to as the RAB5 to RAB7 switch. Differences in endosomal RAB5 and RAB7 mRNA expression have been reported between COVID-19-positive and -negative patients [48]. These small GTPases play a key role in the host cell during coronavirus infection and have been suggested as a potential target for coronavirus vaccine adjuvant [49].

LSAMP (limbic system-associated membrane protein, IGLON3, LAMP) is a neuronal surface glycoprotein expressed in limbic regions and in areas subserving cognition, learning and memory, and autonomic behaviors. The loss of LSAMP leads to reduced expression of the hippocampal mineralocorticoid receptor (MR) [50]. Furthermore, LSAMP can stimulate MR expression, which in turn promotes IL-6 expression [51]. Polymorphic variants of LSAMP have also been associated with left main coronary artery disease [52].

PIKFYVE (phosphoinositide kinase, FYVE-type zinc finger containing) was identified as the target of apilimod, which is a small-molecule inhibitor of IL-12/IL-23 [53]. PIKFYVE is a phosphoinositide 5-kinase that synthesizes PtdIns5P and PtdIns(3,5)biphosphate. Apilimod has a blocking effect on the entry of SARS-CoV-2 into host cells and was considered in a phase II efficacy trial as a COVID-19 treatment. However, this compound actually worsened disease in a COVID-19 murine model [54]. Nevertheless, a novel PIKFYVE inhibitor, XMU-MP-7, effectively over-rides SARS-CoV-2 and its variants in vitro, including the more severe delta and Omicron variants [11]. Complete protection from SARS-CoV-2 lung infection was attained in mice through combined intranasal delivery of PIKFYVE kinase and TMPRSS2 protease inhibitors [55]. Intrinsically, PIKFYVE is found to be involved in influenza and RSV infections, and its inhibitors are promising for a pan-viral approach against respiratory viruses [56].

ADGRL4 (adhesion G protein-coupled receptor L4, ELTD1) is a highly conserved, angiogenesis-associated orphan adhesion GPCR. It has been associated with cardiac and renal function, glioblastoma, and colorectal cancer [57]. Elevated expression of ADGRL4 promotes endothelial-sprouting angiogenesis, without activating canonical GPCR signaling [58]. ADGRL4/ELTD1 is present in extracellular vesicles from endothelial cells, where it occurs as a cleaved extracellular domain that induces angiogenesis in vivo [59]. A comprehensive analysis of the brain transcriptomic profile in response to infection with different SARS-CoV-2 variants found that ADGRL4/ELTD1 was one of five downregulated genes common to all variants. Vaccination with VSV-DG-spike prevented dysregulation of this gene in the K18-hACE2 mouse model [60].

DSC3 (desmocollin 3, DSC3, CDHF3, DSC) belongs to a subfamily of cadherins and is a major component of desmosomes in the keratinocytes of stratified epithelia such as the epidermis. It is also an autoantigen in pemphigus vulgaris [61]. DSC3 was reported to be one of the genes that may be epigenetically modulated by SARS-CoV-2 in the host cell [62].

NUDCD2 (NudC domain-containing 2, NUDCD2, NudCL2) is a co-chaperone that functions in concert with Hsp90 [63]. NUDCD2 interacts with and stabilizes HERC2 [64], which is a large E3 ubiquitin ligase with multiple structural domains. It has been implicated in an array of cellular processes, such as COPI-coated vesicle budding and vesicle-mediated transport/localization [65]. NUDCD2 interacts with the SARS-CoV-2 spike protein and shows increased ubiquitination following SARS-CoV-2 infection [66].

NECTIN2 (nectin cell adhesion molecule 2, CD112) is a high-affinity ligand for DNAM-1 (activation) receptors and a low-affinity ligand for TIGIT (inhibition) receptors on the surface of natural killer (NK) cells. NK cells are critical effectors of antiviral immunity. In patients with severe COVID-19, infected cells and monocytes upregulate the surface expression of NECTIN2/CD112. The DNAM-1 receptor is subsequently internalized following ligation, whereas TIGIT inhibits DNAM-1 expression at the cell surface. This leads to the exhaustion of NK cells, thus allowing escape of SARS-CoV-2 from NK cell killing [67]. Furthermore, the blocking of TIGIT led to increased killing of SARS-CoV-2-infected Calu-3 cells by healthy NK cells [68].

XXYLT1 (xyloside xylosyltransferase 1) is a retaining glycosyltransferase that negatively regulates Notch receptor activation by adding xylose to the Notch extracellular domain. Loss of the Xxylt1 homologue in Drosophila promotes delta-mediated activation of Notch [69]. XXYLT1 is hypermethylated in lung cancer and shows lower mRNA expression levels, especially in male patients [70].

PRKAA2 (protein kinase AMP-activated catalytic subunit alpha 2, AMPKa2) is one of two isoforms of the α-subunit of AMPK (AMP-activated protein kinase). AMPK is a heterotrimeric protein complex formed by α, β, and γ subunits and is active under conditions of low cellular energy. After binding AMP and ADP, the net effect of AMPK activation is the stimulation of glucose uptake, lipogenesis and triglyceride synthesis, inhibition of cholesterol synthesis, and modulation of insulin secretion by pancreatic β-cells. AMPK also has a regulatory role in the immune system [71]. SARS-CoV-2 infection dysregulates the renin–angiotensin system due to ACE2 internalization and degradation, but AMPK phosphorylates ACE2 Ser680 to stabilize ACE2. Endothelial dysfunction was observed in mice with endothelial cell-specific deletion of AMPKa2 or ACE2 [72].

SETBP1 (SET binding protein 1) stabilizes SET protein by blocking its cleavage by proteases. SET is a multifunctional protein that acts as a cancer-promoting factor and inhibitor of protein phosphatase 2A (PP2A), a major serine/threonine protein phosphatase [73]. PP2A is involved in hepatic gluconeogenesis, probably via PP2A-AMPK-FoxO1 signaling [74,75]. SETBP1 gene/protein was investigated in chicken coronavirus, an infectious bronchitis virus [76].

PPP1R26 (protein phosphatase 1 regulatory subunit 26, KIAA0649, NRBE3) activates glycolysis in hepatocytes by enhancing PKM2 (pyruvate kinase M2) splicing [77]. PKM2 promotes Th17 cell differentiation and autoimmune inflammation by fine-tuning STAT3 activation [78]. As part of the inflammatory response to severe COVID-19 illness, neutrophils display increased cytosolic PKM2 [79].

CPSF2 (cleavage and polyadenylation-specific factor 2, CPSF100) is a subunit of the CPSF complex, which is the core component of 3′ end RNA processing. This complex specifically recognizes the hexameric AAUAAA poly(A) signal (PAS) that defines the pre-mRNA processing site, cleavage, and polyadenylation [80]. Alternative polyadenylation of innate, immune-related mRNAs is observed in COVID-19 patients [81]. Moreover, CPSF2 has been identified amongst the top-30 hub genes that underlie the pathophysiological link between acute myocardial infarction and COVID-19 [82].

### 2.3. Genes/Proteins Identified by 1-L Transcription of C-(AA)n-N Sequences 

**Figure 4:**
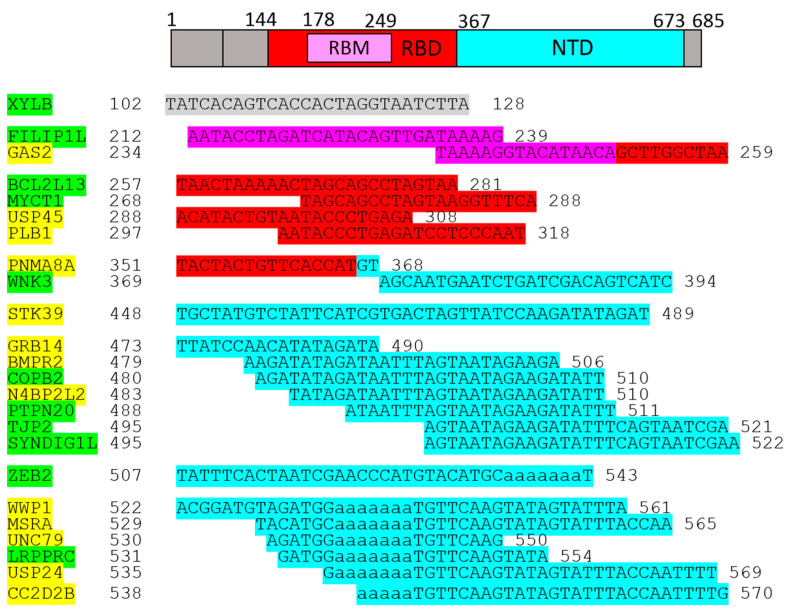
Identified genes/proteins by 1-L transcription of *C*-(AA)n-*N* sequence. Schematic domain architecture of reversed S1 subunit sequence. Green highlights show alignments with the gene transcript RNA sequence (post-transcriptionally repressed); yellow highlights show alignments with the reverse complement RNA sequences (post-transcriptionally promoted).

The identified genes are displayed in Figure 4 according to the alignments.

XYLB (xylulokinase) catalyzes the ATP-dependent phosphorylation of D-xylulose to produce xylulose 5-phosphate (Xu5P), which is a key regulator of glucose metabolism and lipogenesis [83]. In the presence of excess glucose, Xu5P activates PP2A phosphatase, which in turn activates phosphorylated ChREBP. This TF then mediates glucose-induced lipogenesis [83]. Xu5P is involved in SARS-CoV-2 infection [84]. Because its level decreases sharply in the plasma of COVID-19 patients, it was proposed as a potential metabolomic biomarker [85,86]. XYLB was also identified among the top hypermethylated genes [87].

FILIP1L (filamin A interacting protein 1) promotes the ubiquitination and degradation of heat shock factor 1 (HSF1) through the ubiquitin–proteasome system [88]. HSF1 is the master regulator of HSP90 in cells, and HSF1-mediated transcription is primarily responsible for upregulating HSP90 [89]. HSP90 is essential for SARS-CoV-2 viral replication [13,90]. Human coronaviruses activate and hijack the proteostasis guardian HSF1 to enhance viral replication [91], and FILIP1L is downregulated in COVID-19 [92]. 

GAS2 (growth arrest specific 2) is involved in an array of biological processes, including cytoskeletal reorganization, the cell cycle, apoptosis, cancer development, and the promotion of senescence [93]. Up to 80% of patients that survive acute respiratory distress syndrome (ARDS) secondary to SARS-CoV-2 infection present with persistent anomalies in pulmonary function after hospital discharge. Whole-blood transcriptome profiling revealed decreased expression of GAS2 in patients with severe diffusion impairment [94].

BCL2L13 (BCL2-like 13, BCL-RAMBO) is an atypical member of the BCL2 family that regulates apoptosis, mitochondrial fragmentation, and mitophagy [95]. ACE2 stimulates the induction of browning in white adipose tissue [96], while the expression of BCL2L13 increases during white adipose tissue browning [97]. COVID-19 is known to instigate adipose browning and atrophy [98]. The SARS-CoV-2 protein Nsp14 is involved in viral RNA replication and immune escape. It alters gene expression mostly at the transcriptional level and downregulates the expression of BCL2L13 [99].

MYCT1 (MYC target 1, MTLC) regulates the translational efficiency of glycogen enzymes, thereby altering the glycogen shunt in a RACK1-dependent manner. Downregulation of MYCT1 decreases GSK3A expression, which in turn promotes glycogen synthesis [100]. The MYCT1 promoter region contains a vitamin D receptor (VDR) binding site, and VDR transcriptionally upregulates MYCT1 [101]. In addition, MYCT1 facilitates cross-talk between angiogenesis and immunity within the tumor microenvironment [102].

USP45 (ubiquitin specific peptidase 45) is a de-ubiquitinase for MYC/c-Myc [103] and correlates negatively with the infiltration of NK cells, Th1 cells, macrophages, and dendritic cells into the tumor microenvironment [104]. 

PLB1 (phospholipase B1) is an integral membrane enzyme that can remove fatty acids from both the sn-1 and sn-2 positions of glycerophospholipids. PLB1 expression has been reported in several mammalian tissues, including the intestine, epididymis, epidermis, and testis [105]. Its precursor has also been purified from normal human granulocytes, suggesting a possible role in the generation of lipid mediators of inflammation [106].

PNMA8A (paraneoplastic Ma family member 8A, PNMAL1 paraneoplastic Ma Antigen-Like 1) is a relatively uncharacterized PNMA family member found only in humans and chimpanzees [107]. PNMA family members are associated with paraneoplastic disorder, whereby the tumor immune response breaks immune tolerance and starts to attack normal tissue. PNMAL1 was suggested as a potential prognostic biomarker of human PDAC (pancreatic ductal adenocarcinoma), since elevated expression was significantly associated with better overall survival [108]. Furthermore, PNMA8A/PNMAL1 was listed among the candidate genes for stroke [109], as well as among cytotoxicity-related genes in CD4^+^ and CD8^+^ T cells that mark progression to type 1 diabetes [110].

WNK3 (WNK lysine-deficient protein kinase 3, with-no-lysine kinase 3) is a positive regulator of NKCC2 and NCC, which are renal cation-Cl^−^ cotransporters required for normal blood pressure homeostasis. The WNK3–SPAK–NKCC1 phosphorylation cascade in the aorta is regulated by dietary salt intake and is physiologically important for vasoconstriction by angiotensin II [111]. Angiotensin II activates the phosphorylation cascade through the angiotensin II type 1 receptor (AT1R) [111]. WNK3 localizes in vascular smooth muscle cells (VSMCs) undergoing cell division. A high-glucose medium increases WNK3 signaling in VSMCs undergoing mitosis, which could explain the increased thickness of aortic tissues in subjects with diabetes [112]. In the heart, CUL3 cooperates with adaptors such as KLHL2 and RHOBTB1 to specifically recognize substrates such as WNK3 and PDE5 for ubiquitination and subsequent proteasomal degradation [112]. Interestingly, WNK3 is the host protein that interacts with SARS-CoV-2 RNA [113].

STK39 (serine/threonine kinase 39, DCHT, PASK, SPAK) showed a strong signal in a GWAS meta-analysis of hospitalized COVID-19 patients with severe disease [114]. An earlier whole-genome association study also identified STK39 as a hypertension susceptibility gene [115]. MiR-223-3p-loaded exosomes from bronchoalveolar lavage fluid reduced acute lung injury by inhibiting the expression of STK39 and promoting alveolar macrophage autophagy [116].

GRB14 (growth factor receptor-bound protein 14) is a negative regulator of insulin receptor (INSR). GRB14 associates with INSR and inhibits its tyrosine kinase activity. This represses post-receptor insulin signaling by preventing the activation of insulin receptor substrate-1 (IRS1) [117]. Insulin resistance increases the risk of developing type 2 diabetes (T2D), and the inhibition of GRB14 may improve glucose homeostasis in T2D [118].

BMPR2 (bone morphogenetic protein receptor type 2) encodes a TGFβ type II receptor. This acts as a gatekeeper to protect endothelial cells from elevated responses to transforming growth factor-beta (TGFβ), with its deregulation causing diseases such as pulmonary arterial hypertension (PH) [119]. BMPR2 deficiency in endothelial cells (ECs) does not abolish canonical TGFβ-SMAD2/3 and lateral TGFβ-SMAD1/5 signaling responses but, instead, favors the formation of mixed-heteromeric receptor complexes comprising BMPR1/TGFβR1/TGFβR2 that enable enhanced cellular responses towards TGFβ [119]. TGFβ signals induce epithelial-to-mesenchymal transition (EMT), so that the promotion of BMPR2 will reduce EMT, whereas BMPR2-deficient ECs switch from E-Cadherin to N-Cadherin (EMT) [119]. BMPR2 deficiency helps to recruit lymphocytes, macrophages, and neutrophils to vessels by increasing vascular cytokine production. Moreover, the production of both endothelial GM-CSF and macrophage IL-6 increases in response to reduced BMPR2 [120]. Unfortunately, IL-6 activates signal transducer and activator of transcription 3 (STAT3) to increase the expression of microRNA (miR) 17-5 and miR-20. These bind to the 5′UTR of BMPR2 mRNA to further reduce its expression [121]. Interestingly, the loss of BMPR2 drives NK cell deficiency [122]. SARS-CoV-2 infection promotes pulmonary vascular remodeling and vasoconstriction, which are hallmarks of pulmonary arterial hypertension [123].

COPB2 (COPI coat complex subunit beta 2, β′-COP) is a subunit of the Golgi coatomer complex I (COPI). This consists of seven subunits: α-COP, β-COP, β′-COP, γ-COP, δ-COP, ε-COP, and z-COP. COPI is necessary for retrograde trafficking from the Golgi to the endoplasmic reticulum (ER). COPB2 was proposed as an early predictor of COVID-19 severity, and its level in circulating extracellular vesicles (EVs) is substantially higher in patients with mild infection symptoms compared to those with severe infection [124]. The cytoplasmic tail of the SARS-CoV-2 spike binds to both COPI and COPII vesicle coats. Binding to COPII allows for exit from the ER, while binding to COPI in the early Golgi allows for transit to the cell surface, where it accumulates and can direct the formation of multinucleate syncytia [125]. COPB2 is thought to be an important oncogene in several cancer types, and its downregulation inhibits cell proliferation, induces G1 phase arrest, and induces the synthesis of mRNA and proteins [126].

N4BP2L2 (NEDD4 binding protein 2 like 2, PFAAP5) is a nuclear protein that causes the neutrophil elastase-PFAAP5-Gfi1 repressor complex to repress the neutrophil elastase gene (ELA2). This modulates the production of neutrophils, which are essential for host innate immunity [127]. Neutrophil elastase is one of four serine proteases stored in the azurophil granules of neutrophils. In addition to its role in the degradation of the extracellular matrix, neutrophil elastase also functions as a negative regulator of the inflammation process by degrading bacterial virulence factors and proinflammatory mediators like IL-1β and TNF-α [128]. Interactome analysis of SARS-CoV-2 revealed that N4BP2L2 interacts directly with NSP13, a highly conserved helicase associated with the suppression of IFN production and signaling [129]. N4BP2L2 also appears to be associated with the risk of coronary artery disease [130].

PTPN20 (protein tyrosine phosphatase non-receptor type 20) belongs to a large family of protein tyrosine phosphatases that play a crucial role in cell signal transduction by catalyzing dephosphorylation. PTPN20 was identified as an innate immunity-related gene in gastric cancer with *Helicobacter pylori* infection. The expression of PTPN20 was found to be negatively correlated with the level of M1 macrophages. These cells release cytokines, such as IL-6, IL-12, IL-8, and TNF, that stimulate type 1 T-helper cells [131]. PTPN20 deletion in the choroid plexus epithelium was shown to cause an overproduction of cerebrospinal fluid, contributing to the formation of hydrocephalus [132]. PTPN20 was also listed among the 10 proteins with the highest mean autoantibody response in COVID-19 patients [133].

TJP2 (tight-junction protein 2, zonulae occludentes 2, ZO-2) belongs to a family of ubiquitous scaffold proteins. These provide a structural basis for the assembly of multiprotein complexes at the cytoplasmic surface of the plasma membrane that link transmembrane proteins to the filamentous cytoskeleton. In addition to its structural function, TJP2 is also a regulator in the Hippo pathway, with SARS-CoV-2 known to block IFN signaling through the Hippo pathway [134]. Inactivation of TJP2 leads to the upregulation of YAP [135], which is a negative regulator of innate immunity against a range of viruses [136].

SYNDIG1L (synapse differentiation-inducing 1 like, SYNDIG2, IFITMD4, TMEM90A) has an unknown function, but its homolog SYNDIG1 is a type II transmembrane protein that regulates AMPA receptor content at developing synapses [137]. SYNDIG1L also belongs to the IFN-induced transmembrane protein (IFITM) family of restriction factors, with a broad spectrum of viral inhibition [138].

ZEB2 (zinc finger E-box binding homeobox 2) is a TF required to maintain tissue-specific identities of macrophages. ZEB2-deficient macrophages are outcompeted by wild-type counterparts [139]. This essential TF also plays a key role in hematopoietic lineage specification [140]. For example, ZEB2 promotes the terminal differentiation of CD8^+^ effector and memory T-cell populations during infection [141]. ZEB2 is also a master regulator of EMT and mediates trophoblast differentiation [142,143].

WWP1 (WW domain-containing E3 ubiquitin protein ligase 1, AIP5, Tiul1, hSDRP1) is overexpressed during SARS-CoV-2 infection and is directly involved in the virus life cycle. Moreover, it physically interacts with, and ubiquitylates, the S protein [144]. I3C (Indole-3-carbinol), a natural WWP1 inhibitor from Brassicaceae, displays potent antiviral effects and inhibits viral egress [145]. This E3 ubiquitin ligase also regulates ciliary dynamics via the Hedgehog receptor Smoothened (Smo) [146]. Dysregulation of the Hedgehog pathway is one of the molecular mechanisms underlying COVID-19-induced pulmonary fibrosis [147]. During the early phase of ischemic myocardial infarction (MI), WWP1 expression is upregulated in cardiomyocytes located at the infarct border. Following MI, WWP1 triggers excessive cardiomyocyte inflammation by targeting KLF15 to catalyze K48-linked polyubiquitination and degradation [148]. WWP1 also promotes atypical K27-linked ubiquitin multichain assembly on DVL2 and exacerbates cardiac hypertrophy via the DVL2/CaMKII/HDAC4/MEF2C pathway [149].

MSRA (methionine sulfoxide reductase A) is important in protein antioxidant defense. Methionine residues react with oxidizing species that oxidize methionine to methionine sulfoxide (MetSO). MSRA then catalyzes the reduction of the sulfoxide back to methionine. The non-myristoylated form of MSRA is present in mitochondria, whereas the myristoylated form is a late endosomal protein [150]. Reversible oxidation may be a conserved mechanism that many viruses, including SARS-CoV-2, use to regulate viral polyprotein processing, particularly during oxidative stress [151]. Decreased levels of MetSO were observed in COVID-19 patients compared to influenza or RSV [152]. SARS-CoV-1 increases MSRA mRNA during infection, but SARS-CoV-2 does not [153]. Instead, it appears that SARS-CoV-2 promotes MSRA post-transcriptionally.

UNC79 (unc-79 homolog, NALCN channel complex subunit) is a massive HEAT-repeat protein, important for the localization and function of NALCN-channelosome (Na^+^-leak channel). UNC79 forms an intertwined, anti-parallel superhelical assembly with UNC80, which docks intracellularly to the NALCN–FAM155A pore-forming subcomplex [154]. Extracellular Ca^2+^ regulates neuronal excitability by controlling the size of the NALCN-dependent Na^+^-leak current. Knockout of NALCN causes lethal disruption of the respiratory rhythm in mice, while NALCN or UNC79 mutations in humans cause progressive mental, motor and visual deterioration, and early death [155]. NALCN is a hub gene that is upregulated in COVID-19 [156].

LRPPRC (leucine-rich pentatricopeptide repeat-containing) is a global RNA chaperone that forms a complex with its protein partner SLIRP. This complex stabilizes the mitochondrial transcriptome and exposes sites required for polyadenylation, stabilization, and translation [157]. A subset of mRNAs is exported from the nucleus to the cytoplasm by CRM1 (also known as Exportin 1 or XPO1), which interacts with the NES (nuclear export signal) of RNA export adapter proteins, such as LRPPRC or ELAVL1. The LRPPRC adaptor interacts with 4E-SE (eIF4E-sensitivity element)-containing mRNAs and exports these to the cytoplasm. The 4E-SE-containing mRNAs are recognized by nuclear eIF4E (eukaryotic translation initiation factor 4E) at the 7-methyl guanosine cap of the 5′-end [158]. mRNA export is an important regulatory step in eukaryotic gene expression. Viruses, including SARS-CoV-2, attack and hijack the host mRNA export machinery to suppress gene expression and, thus, the immune/antiviral response [158,159]. RNA export adapter proteins such as LRPPRC or ELAVL1 also function as readers of RNA N6-methyladenosine modification (m6A) [160], which is crucial for the transmission and pathogenicity of COVID-19 [161]. m6A is not only essential for gene expression, translation, and regulation but also has important physiological functions in various aspects of the immune response. However, there is still a paucity of research on associations between m6A and the immune responses during COVID-19 infection [161].

USP24 (ubiquitin-specific peptidase 24, PARK10) is a de-ubiquitinase (DUB) that regulates the activation of TANK-binding kinase 1 (TBK1) [162]. TBK1 is a key signaling component in the production of type I interferons. These have essential antiviral activities, including against SARS-CoV-2 [163]. USP24 interacts with TBK1 to reduce the K63-linked polyubiquitination of TBK1, thereby affecting IRF3 activation and IFN-I production [162]. USP24 increases IL-6 transcription in M2 macrophages by stabilizing p300 and β-TrCP [164]. The PARK10 gene USP24 is also a negative regulator of autophagy [165].

CC2D2B (coiled-coil and C2 domain-containing 2B) has no known function. Global α-cell-like transcriptional reprogramming occurs in Pdx1-deleted islet β cells, with CC2D2B being one of the four most differentially expressed genes in these cells [166].

## 3. Discussion

### 3.1. GEMIN5 as an Example of How Protein Primary Structure Is Involved in Protein–RNA Recognition

The SMN complex (SMN, GEMIN2-8, and UNRIP) drives structural changes in human spliceosomal small-nuclear RNAs. GEMIN3 is the essential helicase, while GEMIN5 acts as the essential “identifier” of the RNA substrate. To demonstrate 1-L transcription and how the protein primary structure is involved in protein–RNA interactions, 1-L-transcribed GEMIN5 is shown here to be compatible with the nucleotide sequence of RNU2-1 (Figure 2). The 1-L-transcribed GEMIN5 nucleotide sequence matches perfectly with the 5- to 28-nucleotide sequence of RNU2-1. This region forms stem 1 in the RNU2-1 secondary structure. It is stable during the structural rearrangement pathway that opens the structured Sm site to make it available for Sm proteins (the predicted pre-snRNA folding pathway [18]). The C-terminus of GEMIN5 matches the Sm site (Figure 2B,D; red), while the N-terminal WD40 domain contains sequences compatible with the U2 Sm site reverse complement RNA sequence (Figure 2; yellow). GEMIN5 has been reported to specifically bind to the m^7^G cap and the Sm site of pre-snRNAs [20]. It, therefore, makes sense that this compatibility is identified in the primary sequence of the protein responsible for binding and delivering snRNAs to the SMN complex. Interestingly, recognition of the U2 Sm site reverse complement RNA sequence suggests interactions with miRNAs that are capable of blocking the Sm site, such as hsa-miR-519e-5p (Figure 2D). MiRNAs are small endogenous RNAs that pair and bind to mRNA sites to induce post-transcriptional repression. Reducing the level of miRNAs or other small regulatory RNAs could, therefore, promote translation. In light of this, alignments with reverse complement RNA sequences (plus/minus strand) are considered promotive (yellow in the figures), while alignments with the RNA sequence of the gene (strand plus/plus) are considered repressive (green in the figures). 

### 3.2. 1-L Transcription of the SARS-CoV-2 Spike Protein S1 Subunit and Genes/Proteins Identified as Being Relevant to COVID-19

Using the 1-L transcriptional approach, 20 of the identified genes/proteins may be directly associated with COVID-19 (Section 3.2.1). Another 17 may be indirectly associated with COVID-19 (Section 3.2.2), while, currently, only 7 cannot be associated with COVID-19 (Section 3.2.3). This finding provides general support for the 1-L transcription methodology.

#### 3.2.1. Genes/Proteins Known to Be Related to COVID-19

The genes/proteins identified by 1-L transcription of the S1 subunit *N*-(AA)n-*C* sequence are listed in Figure 3. PARG, BCAP29, ZYG11B, USP46, ZNF385D, PIKFYVE, ADGRL4, DSC3, NECTIN2, and CPSF2 were directly linked to SARS-CoV-2 infection. 

Increased PAR hydrolase activity by PARG and SARS-CoV-2 Nsp3 is required for IFN antagonism and efficient virus replication [26,27]. Increased levels of ADP-ribose product over-activate TRPM2 channels, leading to intracellular Ca^2+^ overload and a form of programmed cell death [28]. A mendelian randomization analysis of the association between SARS-CoV-2 infection and blood constituents found consistent evidence that COVID-19 is causally associated with BCAP29 [30]. SARS-CoV-2 ORF10 increases the overall E3 ligase activity of the CUL2 complex by interacting with promoted ZYG11B. This increases proteasome-mediated degradation of IFT46 (intraflagellar transport 46), thereby impairing cilia functionality and, hence, pulmonary clearance [12,32]. Machine learning methods revealed that USP46 was amongst the genes identified during sequential vaccination with ChAdOx1/BNT162b2 [35]. ZNF385D was found in an epigenome-wide association study (EPICOVID) [43] and appears as the upstream regulator of MYBL2. ZNF385D and MYBL2 were both listed as hub genes in a study examining the influence of COVID-19 on ischemic stroke [40,41]. Complete protection from SARS-CoV-2 lung infection in mice was achieved through the combined intranasal delivery of PIKFYVE kinase and TMPRSS2 protease inhibitors [55]. In response to infection with different SARS-CoV-2 variants, ADGRL4/ELTD1 was one of five downregulated genes in the brain transcriptomic profile and common to all SARS-CoV-2 variants. Moreover, vaccination (VSV-DG-spike) prevented dysregulation of this gene in the K18-hACE2 mouse model [60]. DSC3 was reported to be one of the genes that could be epigenetically modulated by SARS-CoV-2 in the host cell [62]. In patients with severe COVID-19, infected cells and monocytes upregulate their surface expression of NECTIN2/CD112, leading to NK cell exhaustion and, hence, to SARS-CoV-2 escape [67]. CPSF2 was identified among the top-30 hub genes underlying the pathophysiological correlation between acute myocardial infarction and COVID-19 [82].

The genes/proteins identified by 1-L transcription of the S1 subunit *C*-(AA)n-*N* reverse sequence are shown in Figure 4. XYLB, FILIP1L, GAS2, BCL2L13, WNK3, STK39, COPB2, N4BP2L2, PTPN20, and WWP1 were directly linked to SARS-CoV-2 infection.

The level of the XYLB product Xu5P decreased sharply in the plasma during SARS-CoV-2 infection [85,86], while XYLB was also identified as one of the top hypermethylated genes [87]. Human coronaviruses can activate and hijack the proteostasis guardian HSF1 to enhance viral replication [91]. SARS-CoV-2 achieves this by suppressing FILIP1L [92], a regulator of HSF1 [88]. GAS2 can promote cell senescence [93]. The whole-blood transcriptome profile revealed decreased expression of GAS2 in patients with severe diffusion impairment [94]. Decreased GAS2 mRNA could be a consequence of GAS2 post-transcriptional promotion (Figure 4). SARS-CoV-2 Nsp14 mediates the effects of viral infection on the host cell transcriptome, with BCL2L13 showing downregulated expression [99]. WNK3 is a host protein that interacts with SARS-CoV-2 RNA [113]. MiR-223-3p-loaded exosomes from bronchoalveolar lavage fluid promote alveolar macrophage autophagy and reduce acute lung injury by inhibiting the expression of STK39 [116]. A GWAS meta-analysis of hospitalized COVID-19 patients found a strong signal for STK39 [114]. COPB2 was proposed as an early predictor of COVID-19 severity [124]. SARS-CoV-2 Nsp13, a highly conserved helicase associated with the suppression of IFN production and signaling, directly interacts with N4BP2L2 [129]. PTPN20 was among the top-10 proteins with the highest mean autoantibody response in COVID-19 patients [133]. WWP1 is overexpressed during SARS-CoV-2 infection and is directly involved in the virus life cycle. Moreover, it physically interacts with the SARS-CoV-2 S protein [144]. 

In summary, 20 of the 44 genes/proteins identified by 1-L transcription of the SARS-CoV-2 spike protein S1 subunit are directly linked to COVID-19, thereby providing support for the 1-L transcription methodology.

#### 3.2.2. Genes/Proteins Indirectly Related to COVID-19

The genes/proteins identified by 1-L transcription of the S1 subunit *N*-(AA)n-*C* sequence are shown in Figure 3. RABEP1, LSAMP, NUDCD2, XXYLT1, PRKAA2, SETBP1, and PPP1R26 were indirectly linked to SARS-CoV-2 infection.

RAB GTPase binding effector protein 1 (RABEP1) is an extended coiled-coil protein with binding sites for RAB5 and RABGEF1, which is the GDP/GTP exchange factor for RAB5 [47]. RAB5 expression increases significantly (*p* < 0.001) in COVID-19 [48]. LSAMP stimulates expression of the hippocampal mineralocorticoid receptor (MR) [50]. This subsequently induces IL-6 expression [51], which is also promoted in COVID-19 [167]. HSP90 facilitates SARS-CoV-2 structural protein-mediated virion assembly and promotes virus-induced pyroptosis [168]. NUDCD2 is a co-chaperone that functions with HSP90 [63]. Repressed XXYLT1-glycosyltransferase negatively regulates Notch receptor activation by adding xylose to the Notch extracellular domain [169]. SARS-CoV-2 infection destabilizes Treg cells through a Notch1-dependent mechanism and promotes systemic inflammation [170]. SARS-CoV-2 infection is characterized by the internalization and degradation of ACE2. The post-transcriptional support of PRKAA2/AMPKα2 (Figure 3) involved in the AMPK phosphorylation-induced stabilization of ACE2 is, therefore, logical [72]. SETBP1 gene/protein was investigated in chicken coronavirus, an infectious bronchitis virus [76]. PPP1R26 activates glycolysis by increasing PKM2 (pyruvate kinase M2) [77]. Furthermore, neutrophils show increased PKM2 during the inflammatory response to severe COVID-19 [79].

Genes/proteins identified by 1-L transcription of the S1 subunit *C*-(AA)n-*N* reverse sequence are shown in Figure 4. MYCT1, PLB1, GRB14, BMPR2, TJP2, ZEB2, MSRA, UNC79, LRPPRC, and USP24 were indirectly linked to SARS-CoV-2 infection.

GSK3 inhibitors impair the replication of SARS-CoV-2 [171], and MYCT1 regulates the translation efficiency of glycogen enzymes such as GSK3A [100]. PLB1 phospholipase cleaves both sn-1 and sn-2 acyl chains and is active in human neutrophils, suggesting a role in the generation of lipid mediators of inflammation [106]. Furthermore, secretory phospholipase A2, which cleaves only the sn-2 acyl chain, is a predictive marker of the severity and outcome of COVID-19 patients [172]. GRB14 is a negative regulator of the insulin receptor [117]. COVID-19 triggers insulin resistance in patients, causing chronic metabolic disorders that were non-existent prior to infection [173]. BMPR2 acts as a gatekeeper to protect endothelial cells from increased TGFβ responses, as highlighted by its deregulation in diseases such as pulmonary arterial hypertension (PH) [119]. SARS-CoV-2 infection promotes pulmonary vascular remodeling and vasoconstriction, which are hallmarks of PH [123]. TJP2 is a regulator in the Hippo pathway, and SARS-CoV-2 is known to block IFN signaling through this pathway [134]. Inactivation of TJP2 is sufficient to upregulate YAP [135], which is a negative regulator of innate antiviral immunity. Lack of ZEB2 leads to the selective loss of terminally differentiated, short-lived effector CD8^+^ T cells [141]. A signature of long-lived CD8^+^ T cells is observed in acute SARS-CoV-2 infection [174]. Promoted MSRA catalyzes the reduction of methionine sulfoxide (MetSO) to methionine. Decreased levels of MetSO are observed in COVID-19 patients, as compared to those with influenza or RSV [152]. Promoted UNC79 is a very large HEAT-repeat protein important for the localization and function of NALCN-channelosome [154]. NALCN is one of 10 upregulated hub genes in COVID-19 [156]. LRPPRC interacts with 4E-SE-containing mRNAs and exports them to the cytoplasm. Viruses attack and hijack the host mRNA export machinery to suppress host gene expression and, hence, the immune/antiviral response [158]. The SARS-CoV-2 NSP1, NSP5, NSP6, NSP16, ORF3a, ORF9b, and E proteins interact with LRPPRC [159]. USP24 de-ubiquitinase is a regulator of TBK1 [162]. The pharmacological inhibition of TBK1 attenuates the immunopathology of SARS-CoV-2 infection [163].

In summary, 17 of the 44 genes/proteins identified by 1-L transcription of the SARS-CoV-2 spike protein S1 subunit are indirectly linked to COVID-19.

#### 3.2.3. Unknown Genes Related to COVID-19

Genes/proteins identified by 1-L transcription of the S1 subunit are shown in Figure 3 and Figure 4. DAW1, ESF1, KCTD2, USP45, PNMA8A, SYNDIG1L, and CC2D2B are novel genes/proteins that may be linked to COVID-19.

Promoted DAW1 appears to be a regulator of the onset of cilia motility [23]. ESF1 was identified as one of five hub genes that modulate angiogenesis, leading to obesity-induced cardiac injury [37]. Knockout of adenylyl cyclase type 5 (AC5) results in a 30% increase in healthy life span, while pharmacological inhibition of AC5 protects against cardiac stress, diabetes, and obesity [45]. KCTD2 blunts the sensitization of AC5 [44], and, hence, the repression of KCTD2 decreases this protection. Promoted USP45 is upregulated in most tumor types and correlates negatively with the infiltration of NK cells, Th1 cells, macrophages, and dendritic cells [104]. Promoted PNMA8A/PNMAL1 was listed among stroke candidate genes [109], as well as among cytotoxicity-related genes in CD4^+^ and CD8^+^ T cells that mark progression to type 1 diabetes [110]. Repressed SYNDIG1L/TMEM90A belongs to IFN-induced transmembrane proteins (IFITMs), a unique family of restriction factors, with a broad spectrum of viral inhibition [138]. CC2D2B is one of the four most differentially expressed genes following global α-cell-like transcriptional reprogramming of Pdx1-deleted islet β cells [166].

### 3.3. Immune Responses and Inflammation 

Post-transcriptionally promoted USP24 (Figure 4) was recently identified as an important regulator of antiviral immunity. This de-ubiquitinase reduces K63-linked polyubiquitination of TBK1. As a consequence, USP24 knockdown improves IFN-I production and dramatically inhibits EV71 infection [162]. Post-transcriptional repression of TJP2 (Figure 4) upregulates YAP [135], which is a mediator in the Hippo pathway and a negative regulator of innate immunity against various viruses [136]. YAP also prevents the K63-linked ubiquitination of TBK1 and disrupts its interaction with IRF3, thereby decreasing virus-induced IFN-I [136]. Interestingly, high glucose levels activate YAP signaling to promote vascular inflammation [175]. It was observed very early that diabetes mellitus and hyperglycaemia are associated with COVID-19 severity and increased mortality [176,177]. On the other hand, in the other pathway, SARS-CoV-2 NSP9 appears to support K63-linked ubiquitination and signaling by TBK1 [178]. Pharmacological inhibition of TBK1 was shown to attenuate immunopathology in a murine model of SARS-CoV-2 infection [163]. While initially protective, sustained engagement of type I interferons is associated with damaging hyper-inflammation in severe COVID-19 patients. Paradoxically, the SARS-CoV-2 spike protein S1 subunit appears to post-transcriptionally inhibit both TBK1 signaling (USP24, TJP2) and IFN-I production, which could confer a positive effect from vaccination. 

Post-transcriptionally promoted USP24 de-ubiquitinase may decrease TBK1 activity by reducing K63-linked polyubiquitin. However, USP24 promotion in M2 macrophages increases IL-6 transcription by stabilizing p300 and β-TrCP [164]. Increased IL-6 is a biomarker in COVID-19 [172]. Promoted LSAMP can induce the expression of the hippocampal mineralocorticoid receptor (MR) [50], and MR stimulation also promotes IL-6 expression [51]. PTPN20 showed a significant negative correlation with the level of M1 macrophages. These release cytokines, such as IL-6, IL-12, IL-8, and TNF, that stimulate type 1 T-helper cells [131]. Promotion of LSAMP (Figure 3), suppression of PTPN20 by the S1 subunit, and the increase in IL-6 highlight the negative effects of vaccination.

Increased IL-6 inhibits BMPR2 via the STAT3–miR-17/92–BMPR2 pathway [121]. Dysfunctional BMPR2 signaling is a key feature of pulmonary hypertension (PH) [120,122]. BMPR2 is a TGFβ type II receptor protein that protects endothelial cells from increased TGFβ signaling [119]. BMPR2 deficiency drives further macrophage IL-6 production and increases endothelial GM-CSF [120]. The S1 subunit post-transcriptionally promotes BMPR2 (Figure 4), which could imply a positive effect of vaccination. IL-6 and TGFβ induce the development of Th17 cells from naïve T cells. In contrast, IL-6 inhibits TGFβ-induced Treg differentiation [179]. Profound perturbations of Treg cells and an uncontrolled inflammatory response are hallmarks of severe COVID-19 [180]. Promoted PPP1R26 (Figure 3) activates glycolysis by enhancing the splicing of PKM2 (pyruvate kinase M2) in hepatocytes [77]. PKM2 promotes Th17 cell differentiation (Th17 defense, the production of IL-22 and IL-17 cytokines and inflammation/autoimmunity) [78]. Interestingly, previous 1-L transcription of the E-protein led to the conclusion that SARS-CoV-2 promotes type 3 immunity against extracellular pathogens (Th17 defense) [13]. 

Phospholipase A2 (PLA2) and IL-6 are predictive markers for the severity of COVID-19 [172]. PLA2 cleaves the sn-2 acyl chain to yield fatty acids and lysophospholipids, leading to the production of a wide variety of mediators that modulate immunity [181] and stimulate SARS-CoV-2 infection [182]. For example, PLA2 contributes to anti-helminth defense by hydrolyzing membrane phospholipids (Th2-defence, production of IL4 and IL5 cytokines, allergy/asthma) [181]. Previous “1-L transcription” revealed that SARS-CoV-2 reorients the immune response to type 2 immunity [10]. On the other hand, PLA2 products represent the first step in the cyclooxygenase (COX) pathway. This leads to the synthesis of prostaglandins, eicosanoids, and thromboxanes involved in inflammation, thereby worsening SARS-CoV-2 infection [182,183,184]. The S1 subunit promotes phospholipase B1 (PLB1, Figure 4), which could similarly facilitate virulence through host cell lysis because it may be involved in the same pathways as PLA2 (KEGG). In addition, the essential hematopoietic TF ZEB2, which attenuates LPS-induced inflammation [185], is repressed (Figure 4). In contrast, N4BP2L2 (Figure 4), which represses neutrophil elastase (ELA2)-mediated degradation of proinflammatory mediators (IL-1β, TNF-α…) [127,128], is promoted.

Promoted PNMA8A/PNMAL1 (Figure 4) is associated with paraneoplastic disorder, when the tumor immune response breaks immune tolerance and begins to attack the normal tissue [108]. On the other hand, PNMA8A/PNMAL1 was also listed among stroke candidate genes [109], and ZNF385D and CPSF2, which are repressed by the S1 subunit (Figure 3), were also associated with inflammation and stroke in relevance to COVID-19 [40,186]. 

The S1 subunit promotes NECTIN2/CD112 (Figure 3). In patients with severe COVID-19, infected cells and monocytes upregulate the surface expression of NECTINs. NECTIN2/CD112 is the high-affinity ligand for DNAM-1 (activation) receptors and low-affinity ligand for TIGIT (inhibition) receptors on the surface of NK cells, which are critical effectors of antiviral immunity. NK cells from patients with severe COVID-19 internalize DNAM-1 following binding to NECTIN2/CD112, whereas TIGIT inhibits NK cells following its low-affinity binding. This mechanism underlies NK cell activation, exhaustion, and dysfunction in severe COVID-19 [67,68]. Promoted USP45 (Figure 4) is upregulated in most tumor types and correlates negatively with the infiltration of NK cells in the tumor microenvironment [104]. USP45 may be involved in NK cell activation, exhaustion, and dysfunction. The aforementioned BMPR2 could also be involved, since its dysregulation facilitates immune escape via NK cell activation, exhaustion, and dysfunction [187,188].

### 3.4. T2D and Cardiac Stress

Type 2 diabetes (T2D) is a form of diabetes mellitus characterized by high blood sugar, insulin resistance, and relative lack of insulin. T2D is known as adult-onset diabetes, with obesity being a major risk factor. A significant association has been found between T2D risk factors or genetic susceptibility to T2D and the severity of COVID-19 [189].

The S1 subunit represses SETBP1 (Figure 3). SETBP1 functions as a stabilizer of the SET protein [73], which is an inhibitor of PP2A. The repression of SETBP1 activates PP2A-FOXO1 signaling, and the key gluconeogenic TF FOXO1 subsequently activates hepatic gluconeogenesis [74]. In addition, PP2A-FOXO1 signaling is activated via the TGFβ1-Smad3 pathway [74]. As noted above, the characteristic increase in IL-6 during COVID-19 inhibits BMPR2 [120], which normally protects against increased TGFβ signaling [119], and upregulated TGFβ1 contributes to hyperglycemia [190]. On the other hand, the S1 subunit is a post-transcriptional promoter of BMPR2 (Figure 4) and supports AMPK by promoting PRKAA2/AMPKa2 (Figure 4). AMPK is thought to be an inhibitor of FOXO1 signaling [74]. In addition, the repression of MYCT1 (Figure 4), which is a switch for the glycogen shunt [100], decreases glycogenolysis. In summary, despite supporting PP2A activity via the repression of SETBP1, vaccination could have a positive effect by reducing gluconeogenesis and glycogenolysis, thereby lowering high blood sugar. 

PDX1 maintains β-cell identity and function by repressing an α-cell program. β-cell-specific removal of PDX1 results in severe hyperglycemia within days [166]. In this model, CC2D2B is downregulated and is one of the four most differentially expressed genes [166]. In what appears to be a positive effect of vaccination, the S1 subunit promotes CC2D2B post-transcriptionally (Figure 4).

Previous 1-L transcription revealed repression of insulin signaling by the SARS-CoV-2 envelope protein E [13]. S1-promoted GRB14 (Figure 4), which is a negative regulator of the insulin receptor (INSR), represses post-receptor insulin signaling and increases insulin resistance [117,118]. This indicates that vaccination directly supports insulin resistance.

Genetic predisposition to obesity may contribute to the risk of T2D and cardiac injury. S1-repressed ESF1 (Figure 3) was identified as one of five hub genes responsible for obesity-induced cardiac injury by affecting angiogenesis in the heart [37]. The highly conserved, angiogenesis-associated orphan adhesion GPCR ADGRL4/ELTD1 is present in extracellular vesicles derived from endothelial cells. It occurs as a cleaved extracellular domain that induces angiogenesis in vivo [59]. ADGRL4/ELTD1 was one of five downregulated genes and was common amongst all SARS-CoV-2 variants [60]. Vaccination could, therefore, have a positive effect. S1 promotes ADGRL4/ELTD1 at the post-transcriptional level (Figure 3).

Knockout of adenylyl cyclase type 5 (AC5) results in a 30% extension of healthy life. Inhibition of AC5 may, therefore, have therapeutic potential not only for cardiac stress but also for aging, diabetes, and obesity [45]. KCTD2 binding to the Gβγ dimer inhibits Gβγ-mediated AC5 sensitization [44]. S1 represses KCTD2 (Figure 3), suggesting that vaccination will have a negative effect.

In the heart, CUL3 cooperates with adaptors such as KLHL2 and RHOBTB1 to specifically recognize substrates such as WNK3 and PDE5 for ubiquitination and subsequent proteasomal degradation [112]. Previous 1-L transcription revealed that E post-transcriptionally promotes RHOBTB1, which subsequently downregulates PDE5 and increases cAMP signaling [13]. Hyperglycemia and hyperinsulinemia increase WNK3 signaling in VSMCs undergoing mitosis, which may explain the increased thickness of aortic tissues in subjects with T2D [112]. However, the S1 subunit post-transcriptionally represses WNK3 (Figure 4), meaning that vaccination could have a positive effect.

WWP1 expression is upregulated in cardiomyocytes located at the infarct border during the early phase of ischemic myocardial infarction (MI). WWP1 triggers excessive cardiomyocyte inflammation after MI [148], as well as exacerbating cardiac hypertrophy [149]. The WW domain-containing E3 ubiquitin protein ligase is overexpressed during SARS-CoV-2 infection and is directly involved in the virus life cycle [144]. The S1 subunit post-transcriptionally promotes WWP1 (Figure 4), so that vaccination in combination with SARS-CoV-2 infection will have a negative effect and may lead to severe cardiac damage. 

### 3.5. Cilia and Lung Injury

Promoted ZYG11B (Figure 3) is a substrate receptor for Cullin 2-RING E3 ubiquitin ligase (CUL2). SARS-CoV-2 ORF10 was shown to increase the activity of the CUL2^ZYG11B^ complex by interacting with ZYG11B. Enhanced CUL2^ZYG11B^ activity causes increased ubiquitination and subsequent proteasome-mediated degradation of the IFT46 protein in the IFT-B complex, thereby impairing both the biogenesis and maintenance of cilia [12,32]. Promoted DAW1/ODA16 (Figure 3) is a cargo adapter. The transport of ciliary proteins and complexes requires adapters that link them to intraflagellar transport (IFT) trains. DAW1/ODA16 physically bridges ODA-proteins with the IFT-B complex protein IFT46 [22]. The consumption of IFT46 by CUL2^ZYG11B^-mediated degradation and DAW1/ODA16 interaction impairs cilia function [12]. Therefore, vaccination in combination with SARS-CoV-2 infection may have a negative effect on pulmonary clearance by cilia and favors other respiratory infections. Moreover, promoted WWP1 (Figure 4) regulates ciliary dynamics via the Hedgehog receptor Smoothened (Smo) [146], and a dysregulated Hedgehog pathway is one of the molecular mechanisms of COVID-19-induced pulmonary fibrosis [147]. 

Epithelial-to-mesenchymal transition (EMT) is a conserved process, during which cells in a mature and adherent epithelial-like state are converted into a mobile mesenchymal state. Epithelial cells use EMT during wound healing in pulmonary epithelial tissue, but EMT is also active in tumor pathology. The ZEB2 TF is a master regulator of EMT and plays a major role in its induction [142,143]. The S1 subunit represses ZEB2 (Figure 4), which may be positive from an anti-cancer point of view but negative with regard to wound healing in pulmonary epithelial tissue. In addition, TGFβ signals induce EMT, so BMPR2 promotion (Figure 4) has the effect of reducing EMT [119].

Acute lung injury (ALI) is a prelude to acute respiratory distress syndrome (ARDS). ALI can arise from multiple etiologies and frequently results in fulminant respiratory failure and death. Promoted STK39 (Figure 4) is involved in ALI, and inhibiting the expression of STK39 reduces ALI [116]. Up to 80% of patients who survive ARDS secondary to SARS-CoV-2 infection present with persistent anomalies in pulmonary function after hospital discharge. Whole-blood transcriptome profiling revealed decreased expression of GAS2 mRNA in patients with severe diffusion impairment [94]. The S1 subunit post-transcriptionally promotes GAS2 (Figure 4).

## 4. Materials and Methods

As shown in Figure 1B, protein primary structures are involved in protein–RNA recognition/interaction. These processes are driven by 1-L and 2-L codes conserved in the amino acid codons, which can be used to identify mRNA and microRNA (miRNA) sequences compatible with genes/proteins that are post-transcriptionally regulated by the RBP under investigation.

### 4.1. 1-L Transcription Procedure

**Figure 5:**
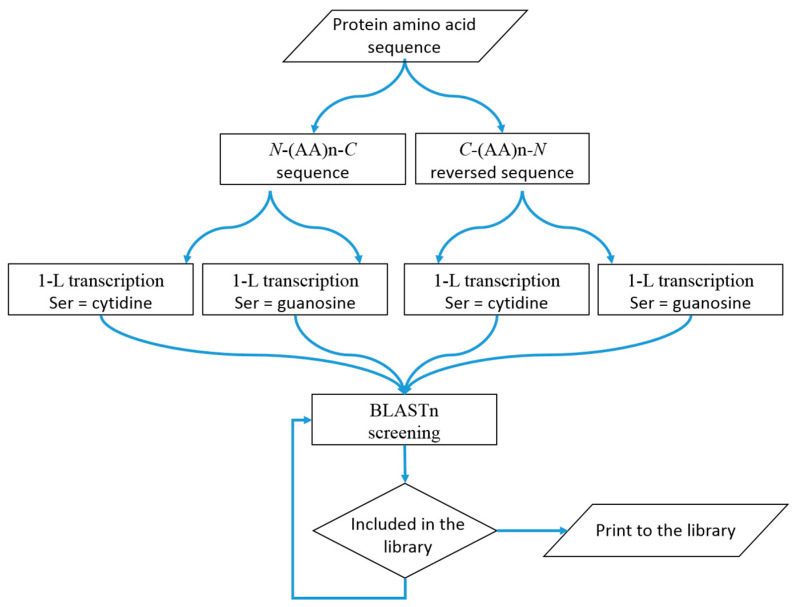
1-L transcription method. A schematic diagram that visually represents the steps and concept.

The 1-L transcription procedure is relatively simple and involves the amino acid sequence of the analyzed RBP being transcribed into an RNA sequence based on the nucleotide at the second position of the amino acid codon (one-letter code). The resulting nucleotide sequence is then used for classical BLASTn screening of the human transcriptome. Reading of the 5′-RNA by RBP can be performed in both directions using the amino acid sequence *N*-(AA)n-*C* or the reverse amino acid sequence *C*-(AA)n-*N* (Figure 1B). Hence, the 1-L transcription should be written for two amino acid sequences: one for *N*-(AA)n-*C* and the other for *C*-(AA)n-*N*. Serine (Ser, S) has two types of codons, one with C (cytidine) at the second position in the amino acid codon and the other with G (guanosine). Thus, for each amino acid sequence, two nucleotide sequences are obtained, one with S-C transcription and the other with S-G transcription. A total of four nucleotide sequences are obtained (Figure 5).

### 4.2. BLASTn Screening Process

BLASTn screening of the human transcriptome was performed as a standard nucleotide blast at NCBI (https://blast.ncbi.nlm.nih.gov/Blast.cgi; accessed on 4 February 2024). This was performed separately for the four nucleotide sequences. The following parameters were used for this search: “Genomic + transcript databases” and “human genomic plus transcript”, “somewhat similar sequences” (blastn), word size = 7, maximum number of target sequences = 500, and expected threshold = 500.

### 4.3. GEMIN5 as an Example

To demonstrate the 1L transcription, a randomly selected RBP GEMIN5 is presented as an example. This RBP was not studied completely here as it was only used as an example to demonstrate how the protein primary structure may be involved in protein–RNA interactions, so only the *N*-(AA)n-*C* direction was applied, and serine was transcribed only as guanosine (Query Sequence). In the case of BLASTn screening process, only one RNA target sequence was applied (>hsa:6066 K14277 U2 spliceosomal RNA, RNU2-1) and entered as Subject Sequence. The following parameters were used for this search: “somewhat similar sequences”, word size = 7, maximum number of target sequences = 500, and expected threshold = 5. However, it is on the user of this method to choose expected threshold higher/lower or change the other parameters, which are considered by the confidence level: “The perfect pairing—low number of hits; imperfect pairing—high number random hits and false positives”. For example, in this case, if expected threshold = 1, then 0 hits; if expected threshold = 5, then 4 hits; if expected threshold = 50, then 19 hits; if expected threshold = 500, then 19 hits.

## 5. Conclusions

Proteins and RNA evolved together in prebiotic history. Primary protein structures, therefore, still conserve basic sequences involved in protein–RNA interactions, as shown by the defined RNA binding protein GEMIN5 (gem nuclear organelle-associated protein 5) and RNU2-1 (U2 spliceosomal RNA). Using the described method, it was shown that 20 of the 44 genes/proteins identified by 1-L transcription of the SARS-CoV-2 spike protein S1 subunit are directly linked to COVID-19 (Section 3.2.1), 17 are indirectly linked to COVID-19 (Section 3.2.2), and 7 genes/proteins cannot currently be associated with COVID-19 (Section 3.2.3). 

By considering the current knowledge on COVID-19, this bioinformatics study showed how vaccination against SARS-CoV-2 infection can change host immune responses and support inflammation (Section 3.3). For example, vaccination will promote NECTIN2/CD112 (Figure 3) on epithelial cells, which is involved in NK cell activation, exhaustion, and dysfunction [67,68]. The essential hematopoietic TF ZEB2, which attenuates LPS-induced inflammation [185], is repressed (Figure 4). In contrast, N4BP2L2 (Figure 4), which represses neutrophil elastase (ELA2) that degrades proinflammatory mediators (IL-1β, TNF-α) [127,128], is promoted. Some of the identified genes/proteins are associated with stroke, diabetes, and cardiac injury. For example, ZNF385D and CPSF2, which are both repressed by the S1 subunit (Figure 3), are associated with COVID-19-related inflammation and stroke [40,186]. S1-promoted GRB14 (Figure 4), which is a negative regulator of the insulin receptor (INSR), represses post-receptor insulin signaling and increases insulin resistance [117,118]. WWP1, which is directly involved in the SARS-CoV-2 life cycle [144], is up-regulated in cardiomyocytes located at the infarct border and triggers excessive cardiomyocyte inflammation [148]. The S1 subunit post-transcriptionally promotes WWP1 (Figure 4), meaning that vaccination in combination with SARS-CoV-2 infection will have a negative effect and may lead to cardiac damage.

In addition, promoted WWP1 (Figure 4) regulates ciliary dynamics via the Hedgehog receptor Smo [146], and a dysregulated Hedgehog pathway is one of the molecular mechanisms underlying COVID-19-induced pulmonary fibrosis [147]. SARS-CoV-2 ORF10 increases the activity of the CUL2^ZYG11B^ complex by interacting with ZYG11B. This mediates the degradation of IFT46, thus impairing the biogenesis and maintenance of cilia [12,32]. The S1 subunit post-transcriptionally promotes ZYG11B (Figure 4); therefore, vaccination in combination with SARS-CoV-2 infection may have a negative effect on pulmonary clearance by cilia and favor respiratory infections.

## Data Availability

Data is contained within the article.
